# ErbB4 signaling stimulates pro-inflammatory macrophage apoptosis and limits colonic inflammation

**DOI:** 10.1038/cddis.2017.42

**Published:** 2017-02-23

**Authors:** Michael A Schumacher, Matija Hedl, Clara Abraham, Jessica K Bernard, Patricia R Lozano, Jonathan J Hsieh, Dana Almohazey, Edie B Bucar, Shivesh Punit, Peter J Dempsey, Mark R Frey

**Affiliations:** 1 The Saban Research Institute, Children's Hospital Los Angeles, Los Angeles, CA 90027, USA; 2 Departments of Pediatrics and of Biochemistry and Molecular Biology, University of Southern California Keck School of Medicine, Los Angeles, CA 90089, USA; 3 Department of Medicine, Yale School of Medicine, New Haven, CT 06510, USA; 4 University of Southern California Herman Ostrow School of Dentistry, Los Angeles, CA 90089, USA; 5 Department of Pediatrics, University of Colorado Medical School, Aurora, CO 80045, USA

## Abstract

Efficient clearance of pro-inflammatory macrophages from tissues after resolution of a challenge is critical to prevent prolonged inflammation. Defects in clearance can contribute to conditions such as inflammatory bowel disease, and thus may be therapeutically targetable. However, the signaling pathways that induce termination of pro-inflammatory macrophages are incompletely defined. We tested whether the ErbB4 receptor tyrosine kinase, previously not known to have role in macrophage biology, is involved in this process. *In vitro*, pro-inflammatory activation of cultured murine and human macrophages induced ErbB4 expression; in contrast, other ErbB family members were not induced in pro-inflammatory cells, and other innate immune lineages (dendritic cells, neutrophils) did not express detectable ErbB4 levels. Treatment of activated pro-inflammatory macrophages with the ErbB4 ligand neuregulin-4 (NRG4) induced apoptosis. ErbB4 localized to the mitochondria in these cells. Apoptosis was accompanied by loss of mitochondrial membrane potential, and was dependent upon the proteases that generate the cleaved ErbB4 intracellular domain fragment, suggesting a requirement for this fragment and mitochondrial pathway apoptosis. *In vivo*, ErbB4 was highly expressed on pro-inflammatory macrophages but not neutrophils during experimental DSS colitis in C57Bl/6 mice. Active inflammation in this model suppressed NRG4 expression, which may allow for macrophage persistence and ongoing inflammation. Consistent with this notion, NRG4 levels rebounded during the recovery phase, and administration of exogenous NRG4 during colitis reduced colonic macrophage numbers and ameliorated inflammation. These data define a novel role for ErbB4 in macrophage apoptosis, and outline a mechanism of feedback inhibition that may promote resolution of colitis.

Crohn's disease and ulcerative colitis, collectively known as Inflammatory Bowel Disease (IBD), remain major incurable clinical challenges. The precise etiologies of these diseases are elusive, but it is clear that innate immune responses, including macrophages, have a central role in their pathophysiology.^1, 2^ For example, when Ly6C^+^ macrophages – acutely recruited phagocytic cells of the innate immune response – are depleted or key signaling pathways in macrophages are inhibited, colitis is mitigated in both acute and chronic models.^2, 3, 4^ In response to tissue damage, infection, or other challenge, populations of Ly6C^+^ monocytes/macrophages are recruited from the bloodstream to potentiate inflammation and fight infection.^2, 5^ However, once the challenge is cleared, failure to properly terminate this acute response or shift macrophage activity from inflammatory to repair phenotypes may contribute to chronic inflammation.^6, 7, 8^ Although some stimuli for macrophage apoptosis are known,^9, 10^ few tissue-derived feedback mechanisms limiting pro-inflammatory cells during re-establishment of homeostasis have been described. Therefore, identifying mechanisms that restrict the pro-inflammatory capacity of activated macrophages, or eliminate pro-inflammatory populations after they are no longer needed, could lead to new therapeutic points of intervention to control IBD and other chronic inflammatory diseases.

The ErbB receptor tyrosine kinases – epidermal growth factor receptor (EGFR), ErbB2, ErbB3, and ErbB4 – are important for homeostatic maintenance of the intestinal tract, and defects in ErbB signaling can contribute to the pathophysiology of IBD.^11, 12, 13^ However, study of these receptors in the GI tract has traditionally focused on their role in epithelial biology, including cell proliferation, survival, and migration. Recently, expression of these receptors in immune populations has been reported,^14, 15, 16^ but their function in these cells is largely undefined.

ErbB4, a key cellular receptor for neuregulin growth factors, is induced in the colon in IBD.^17^ Genome-wide association studies link single-nucleotide polymorphisms in ErbB4 to both Crohn's disease and ulcerative colitis.^18^ Interestingly, the specific ErbB4 ligand neuregulin-4 (NRG4) is suppressed in disease,^19^ whereas exogenous NRG4 is protective in rodent models of intestinal inflammation.^19, 20^ Taken together, these results suggest a protective role for the ErbB4 signaling axis, which is disrupted in IBD. However, the mechanism of this connection is not well understood. Enterocyte apoptosis can be directly blocked by NRG4 exposure,^19^ but it is likely that other cell types also contribute to the protective effect in the whole animal. Recent studies show that ErbB4 is expressed by some macrophage populations.^16, 21^ However, ErbB4 expression levels on macrophages recruited to the colon during inflammation, and its function in these cells, have not been tested.

In this study, we report identification of a signaling axis between intestinal tissue and activated macrophages that may be harnessed to drive resolution of inflammation. We found that ErbB4 is selectively induced during pro-inflammatory activation of macrophages, and ErbB4 activation with its specific ligand NRG4 promotes death of these cells by apoptosis. ErbB4 activation with exogenous ligand administration was therapeutic in acute murine colitis. As inflammatory macrophages are central to IBD pathology, identification of an ErbB4-mediated feedback mechanism that limits or alters their activity advances our understanding of innate immune regulation in colitis. This may allow for novel, more effective approaches to disease treatment focused on targeted inhibition of inflammatory cell subsets.

## Results

### Classical activation of macrophages induces, whereas alternative activation inhibits, ErbB4 expression

The ErbB receptor tyrosine kinases have been predominantly investigated for their roles in epithelial cell growth and migration. However, recent studies demonstrated that some members of this family are also present on immune cells, including macrophages.^14, 16^ We have previously shown that ErbB4, the most biochemically distinct member of this receptor family, is induced in inflamed tissue,^19^ but whether it has a role in macrophages has not been addressed.

**Figure 1:**
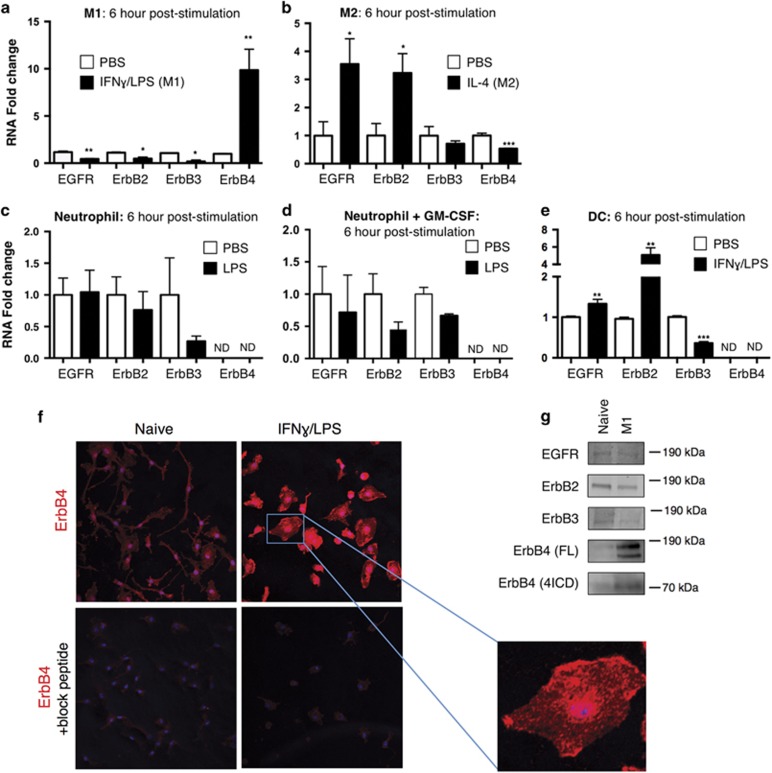
Pro-inflammatory activation of macrophages *in vitro* induces ErbB4 expression. ErbB family member RNA expression was determined after 6 h stimulation of (**a**) BMDMs classically activated with IFN*γ*/LPS, (**b**) BMDMs alternatively activated with IL-4, (**c**) neutrophils activated with LPS, (**d**) neutrophils primed with GM-CSF and then stimulated with LPS, (**e**) BMDCs activated with IFNγ/LPS. For (**a**–**e**), *n*=4–6 independent experiments per group. ND, not detectable. Error bars represent S.E.M. *P<0.05; **P<0.01; ***P<0.001. (**f** and **g**) Unstimulated (naive) or classically activated (M1; 24 h activation shown) BMDMs were subjected to (**f**) immunofluorescence staining (representative of three independent experiments) for ErbB4 (red), nuclei (blue) with or without blocking peptide to primary antibody, or (**g**) western blot analysis (representative blots from *n*=4 independent experiments) for EGFR, ErbB2, ErbB3, ErbB4 (full length, FL; intracellular domain, 4ICD)

Macrophages exist along a continuum of sub-types that perform a variety of pro- and anti-inflammatory functions, as well as tissue repair. To experimentally assess their function *in vitro*, these cells can be driven toward a pro-inflammatory state (classical M1 activation) involved in bacterial clearance, or an anti-inflammatory state (alternative M2 activation) involved in homeostatic and pro-healing responses.^22^ We generated and polarized bone marrow-derived macrophages (BMDM) to M1 and M2 states, and determined the expression pattern of ErbB family members by qPCR. Classical activation with interferon (IFN) *γ*+lipopolysaccharide (LPS) induced ErbB4 10-fold after 6 h, whereas in contrast the other ErbB family members EGFR, ErbB2, and ErbB3 were all significantly decreased (Figure 1a). We also observed induction of ErbB4 mRNA in the immortalized macrophage cell line, RAW267.01, following pro-inflammatory activation with IFN*γ*+LPS (data not shown). To test if this response was specific to pro-inflammatory M1 activation, we also examined macrophages alternatively polarized to an M2 state with interleukin (IL)-4. M2 polarization did not induce ErbB4, but instead resulted in a significant decrease in its expression (Figure 1b). These data suggest that, among macrophage populations, ErbB4 is largely restricted to pro-inflammatory cells.

As other innate immune cells (dendritic cells, neutrophils) can also respond to bacterial cell membrane LPS,^23^ we tested whether induction of ErbB4 is a general feature of TLR4-induced signaling in innate myeloid cells by exposing bone marrow-isolated neutrophils to LPS or bone marrow-derived dendritic cells (BMDCs) to IFN*γ*+LPS. Bone marrow-isolated neutrophils (with or without GM-CSF priming) stimulated with LPS had undetectable levels of ErbB4 (Figures 1c and d). BMDCs stimulated with LPS also displayed a distinct profile of ErbB regulation, and ErbB4 was not detectable in these cells (Figure 1e). This suggests that ErbB4 induction by LPS is a macrophage-specific outcome, rather than a generic TLR4 response.

To confirm ErbB4 induction in M1 macrophages at the protein level, we performed immunofluorescence and western blot analysis. Immunofluorescence staining on LPS-challenged macrophages demonstrated elevated ErbB4 protein expression, both at the plasma membrane and within the cell (Figure 1f). This pattern is consistent with the expression of both full length and proteolytically cleaved intracellular domain (4ICD) forms of the receptor.^24^ ErbB4 protein induction (both full length and 4ICD) was also observed by western blot analysis of naive *versus* M1 macrophages (Figure 1g) and LPS-treated RAW267.01 cells (not shown).

### The ErbB4-specific ligand NRG4 induces pro-inflammatory macrophage apoptosis

**Figure 2:**
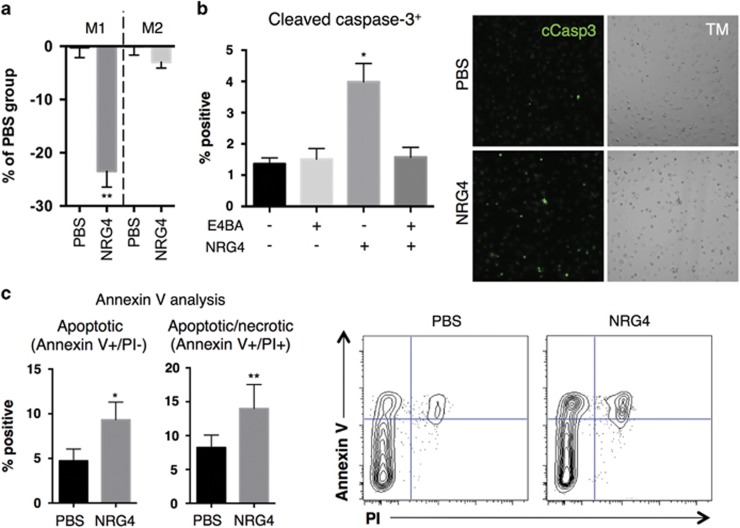
NRG4 induces apoptosis in pro-inflammatory murine macrophages. (**a**) Classically activated (M1) or alternatively activated (M2) BMDMs were treated with NRG4 (100 ng/ml) for 48 h and analyzed by resazurin-based cell titer assay to assess cell numbers compared to control. (**b**) Classically activated BMDMs pre-incubated for 30 min with or without 2 *μ*g/ml ErbB4 neutralizing antibody (E4BA), and treated with or without NRG4 (100 ng/ml) for 48 h, were stained for cleaved caspase-3. TM, transmitted light images of cultures. (**c**) Classically activated BMDMs treated with NRG4 (100 ng/ml) for 48 h were stained for annexin V and propidium iodide (PI), and analyzed by flow cytometry to determine apoptotic cells. *n*=3–6 independent experiments for each panel. Error bars represent S.E.M. *P<0.05; **P<0.01

To determine the role of ErbB4 in pro-inflammatory macrophage biology, we stimulated signaling in these cells using an ErbB4-specific ligand expressed in intestinal tissue, NRG4. Following 48 h treatment, we observed a significant decrease in cell numbers in M1 but not M2 macrophages, indicating that NRG4 selectively inhibits M1 macrophage growth or survival (Figure 2a). LPS activation of macrophages has been reported to halt cell proliferation; we confirmed this in our cultures with EdU staining, and furthermore saw no change in %EdU uptake with or without NRG4 (data not shown), ruling out effects on proliferation. Therefore, we asked whether ErbB4 activation was inducing cell death. NRG4 exposure caused a significant increase in cleaved caspase-3 staining (Figure 2b), indicative of ongoing late-stage apoptosis of these cells. This response was blocked by pre-treatment with ErbB4 neutralizing antibody, demonstrating a requirement for NRG4-ErbB4 binding. As another measure of apoptosis, we performed annexin V analysis. Similar to the cleaved caspase-3 results, annexin V staining revealed a significant increase in apoptosis in response to NRG4 treatment (Figure 2c). These results suggest stimulation of ErbB4 signaling in pro-inflammatory macrophages is a mechanism that limits accumulation of these cells.

### NRG4-induced macrophage death requires protease activity

**Figure 3:**
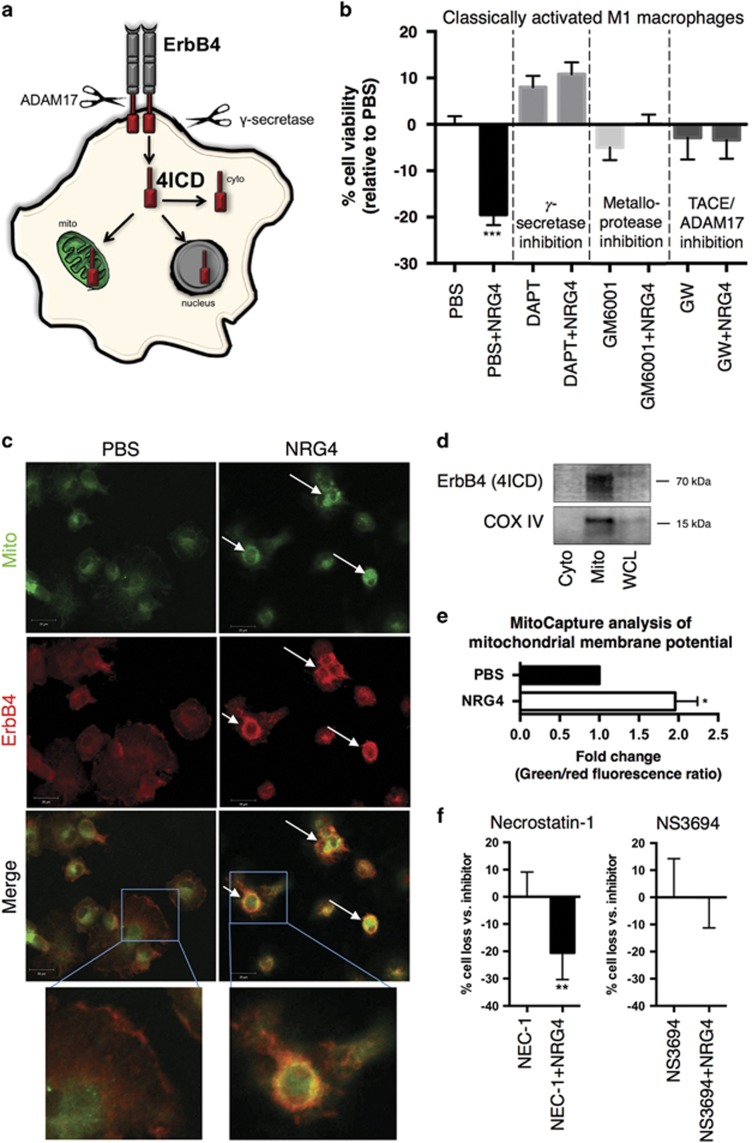
The metalloprotease TACE/ADAM17 and *γ*-secretase are necessary for NRG4-induced macrophage apoptosis. (**a**) Schematic model of potential ErbB4 signaling in macrophages following ligand binding. (Step 1) Extracellular receptor cleavage by ADAM17; (Step 2) intracellular cleavage by *γ*-secretase and generation of ErbB4 intracellular domain (4ICD); (Step 3) migration of the active signaling fragment 4ICD to various intracellular compartments. (**b**) Classically activated BMDMs were pre-treated for 1 h with metalloprotease inhibitor (GM6001, 10 *μ*M), *γ*-secretase inhibitor (DAPT, 10 *μ*M), or ADAM17 inhibitor (GW280264X, 3 *μ*M) followed by 100 ng/ml NRG4 and 100 ng/ml LPS. Percent cell viability was analyzed by rezasurin-based cell titer assay (*n*=5 independent experiments). (**c**) Immunofluorescence analysis of ErbB4 localization to the mitochondria of classically activated BMDMs treated with or without NRG4 (100 ng/ml) for 48 h. Arrows point to representative cells with ErbB4/mitochondrial overlap (images representative from *n*=4 independent experiments). (**d**) Western blot analysis for the ErbB4 4ICD fragment and COX IV (mitochondrial marker) of isolated cytoplasmic and mitochondrial fractions of classically activated BMDMs treated with NRG4 for 48 h. Representative blots from *n*=3 independent experiments shown. (**e**) Classically activated BMDMs were treated with or without NRG4 for 48 h, stained with fluorescent cationic dye (MitoCapture), and analyzed by flow cytometry for red (aggregated dye in healthy mitochondria) and green (cytoplasmic dye resulting from mitochondria with disrupted membrane potential) fluorescence. Fold change in green/red ratio is shown (*n*=5 independent experiments). (**f**) NRG4-induced killing of classically activated macrophages was assessed in the presence of inhibitors to necroptosis (necrostatin-1) or apoptosis (NS3694). Each panel, *n*=5 independent experiments. Error bars represent S.E.M. *P<0.005; **P<0.01; ***P<0.001

Ligand-driven two-step proteolytic cleavage of ErbB4 (by TACE/ADAM17 followed by *γ*-secretase) occurs in some cell types; the resulting soluble 4ICD intracellular domain fragment can localize to the cytoplasm, nucleus, or mitochondria to regulate cellular behavior (Figure 3a). Notably, in breast cancer cells, 4ICD association with mitochondria stimulates apoptosis,^25^ though this has not been observed in non-transformed cells. We used protease inhibitors to test whether this mechanism might have a role in NRG4-induced macrophage apoptosis. Inhibition of either *γ*-secretase (DAPT, 10 *μ*M), broad metalloprotease activity (GM6001, 10 *μ*M), or TACE/ADAM17 (GW280264X, 3 *μ*M) protected against the NRG4-induced cell death (Figure 3b). Consistent with this observation, both immunofluorescence colocalization analysis and western blot analysis of fractionated cells indicate ErbB4 association with mitochondria in macrophages after NRG4 treatment (Figures 3c and d), suggesting the effects of NRG4 may be through 4ICD generation and stimulation of the mitochondrial apoptosis pathway. To clarify a role for the mitochondria, we used Mitocapture cell labeling to assess changes in mitochondrial transmembrane potential (Δ*Ψ*m) of untreated and NRG4-treated M1 macrophages. NRG4 treatment resulted in an increased green/red fluorescence ratio (Figure 3e) in Mitocapture-stained cells indicating decreased Δ*Ψ*m, supporting mitochondrial involvement in NRG4-induced cell loss.

Necroptosis and apoptosis are both forms of programmed cell death that have a role in the macrophage life cycle. To test the involvement of each of these processes in NRG4-induced death, we treated cells with inhibitors against RIPK1 (necrostatin-1, 50 *μ*M) or apoptosome formation/caspase-9 activation (NS3694, 10 *μ*M). Inhibition of necroptosis with necrostatin-1 had no effect on NRG4-induced M1 death, whereas NS3694 abrogated this response (Figure 3f). Taken together, these results suggest that NRG4 induces the mitochondrial apoptosis pathway in M1 macrophages.

### Classically activated human macrophages express ErbB4 and undergo apoptosis in response to NRG4 stimulation

**Figure 4:**
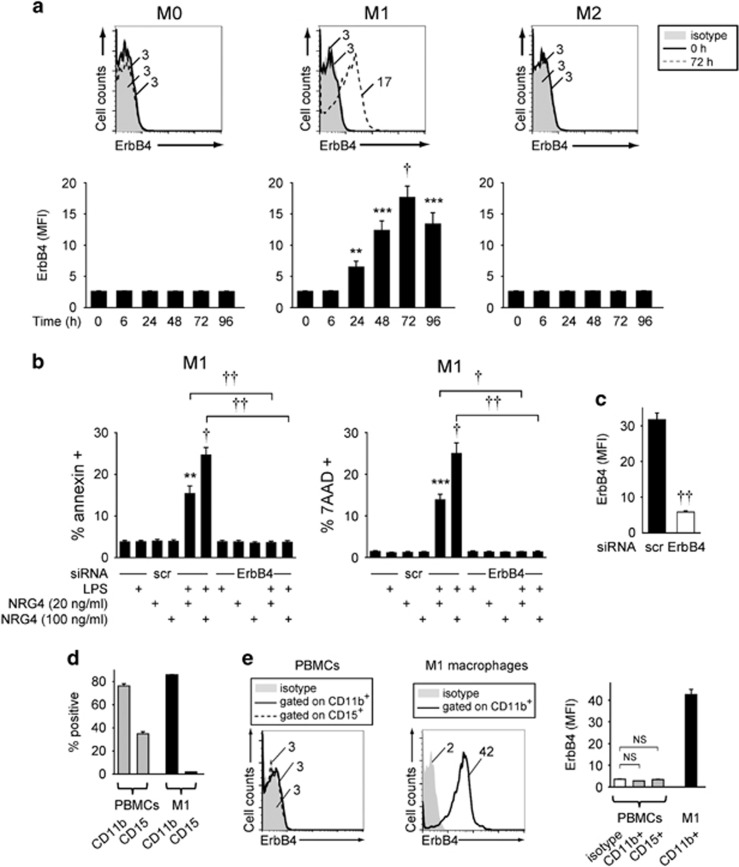
ErbB4 is induced on human monocyte-derived macrophages by pro-inflammatory activation, and mediates NRG4-induced apoptosis. (**a**) Human monocyte-derived macrophages were non-polarized (M0), classically activated (M1), or alternatively activated (M2) (*n*=8 donors), collected at the indicated time points, and stained/analyzed for ErbB4 expression by flow cytometry; representative flow plots and mean fluorescence intensity (MFI) of protein expression are shown. (**b**) Classically activated human macrophages (*n*=8 donors) were transfected with siRNA for ErbB4 and treated with 100 ng/ml LPS+the indicated concentrations of NRG4 for 48 h, and analyzed by flow cytometry for annexin V (apoptosis) and 7AAD (overall cell death). NRG4 treatment was initiated 72 h after M1 polarization at peak ErbB4 expression. Significance was calculated compared to scr siRNA-transfected, untreated cells or as indicated. Similar results were observed at 24 h after LPS+NRG4 treatment in *n*=12. (**c**) ErbB4 knockdown was verified by flow cytometry (*n*=8 donors). (**d** and **e**) Human PBMCs (*n*=4) and M1 macrophages (*n*=4) were stained for CD15 (neutrophil marker), CD11b (myeloid cell marker) and ErbB4, gated on live cells, and analyzed by flow cytometry. (**d**) Percentage of the indicated cell populations expressing CD11b and CD15 shown. (**e**) (Left): Representative histograms showing ErbB4 mean fluorescence intensity (MFI) on the indicated cell populations. (Right): Summary of ErbB4 protein expression. Similar results were seen in an additional independent cohort of *n*=4. Error bars represent S.E.M. *P<0.05; **P<0.01; ***P<0.001; ^†^*P*<1 × 10^−4^; ^††^*P*<1 × 10^−5^

To assess the relevance of our findings to human biology, we generated and polarized macrophages from peripheral blood mononuclear cells (PBMCs) and assessed ErbB4 expression by flow cytometry. Similar to murine cells, pro-inflammatory M1 activation of human macrophages induced ErbB4 expression, assessed here by flow cytometry for protein expression (Figure 4a). Induction was sustained at least 96 h post-stimulation, suggesting capacity to respond to ligand is maintained over time. Alterative M2 activation of these cells had no effect on ErbB4 levels (Figure 4a). Similar to our findings in the mouse, NRG4 exposure elicited a dose-dependent apoptosis of human M1 macrophages as measured by annexin V and 7AAD staining (Figure 4b). Effective ErbB4 knockdown with siRNA (Figure 4c) abrogated this response (Figure 4b), confirming receptor specificity. To clarify the cell type specificity of ErbB4 expression on macrophages, we stained PBMC preparations for the myeloid marker CD11b and neutrophil marker CD15 (Figure 4d), and analyzed gated cells for ErbB4 expression. ErbB4 was not expressed on naive CD11b+ or on CD15+ populations, indicating ErbB4 expression is specific to the M1 macrophage population (Figure 4e). Our findings suggest a conserved role for the ErbB4 signaling axis in macrophage biology between species, and underscore the potential relevance of this feedback mechanism in maintenance of human health.

### ErbB4 is induced during DSS colitis and expressed on Ly6C^+^ inflammatory macrophages

**Figure 5:**
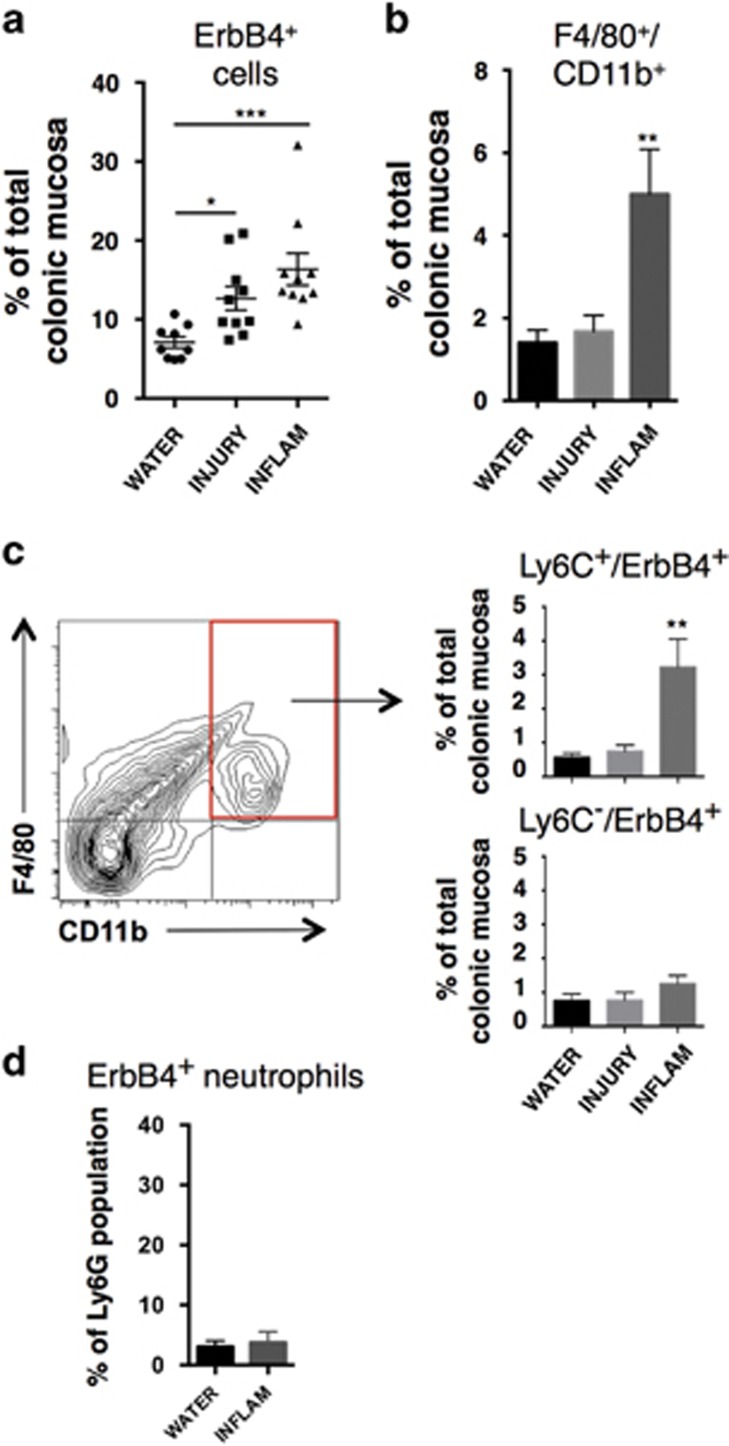
ErbB4 is induced during DSS colitis and is expressed on Ly6C^+^ macrophages. (**a**) Colonic mucosa from mice subjected to DSS colitis were analyzed by flow cytometry to determine percent of cells that are ErbB4+ in colons from mice receiving no DSS (WATER), after 4 days of 3% DSS (INJURY), and after 4 days 3% DSS followed by 3 days without DSS (INFLAM). (**b**) Analysis of F4/80^+^/CD11b^+^ macrophages as a proportion of total mucosal cellularity at indicated time points. (**c**) Ly6C^+^/ErbB4^+^ and Ly6C^−^/ErbB4^+^ populations were analyzed. For (**a**–**c**), *n*=9–10 mice per group from three independent DSS colitis experiments. (**d**) Colonic mucosa from control (WATER) and DSS inflammatory phase (INFLAM) mice were subjected to flow cytometric analysis for Ly6G (neutrophil marker) and ErbB4. Percentage of Ly6G+ cells expressing detectable ErbB4 is shown (*n*=5 mice per condition). Error bars represent S.E.M. *P<0.05; **P<0.01; ***P<0.001

To determine whether macrophage-expressed ErbB4 has a role in intestinal inflammatory disease *in vivo*, we tested whether ErbB4 is expressed on recruited macrophages in the dextran sodium sulfate (DSS) experimental model of murine colitis. In this model, Ly6C^+^ inflammatory macrophage influx is critical for pathogenesis.^2, 26^ Mice were given 3% (w/v) DSS in drinking water for 4 days to elicit acute colonic damage (injury phase), followed by 3 days without DSS (inflammatory phase). Consistent with our previously published findings,^17^ we confirmed an overall increase in ErbB4^+^ cells in the colon by flow cytometric analysis of single cell dissociated mucosa (Figure 5a). Also as expected, numbers of F4/80^+^/CD11b^+^ macrophages in the colon were significantly increased by the inflammatory phase at day 7 (Figure 5b). To characterize ErbB4 expression on these cells, we analyzed the F4/80^+^/CD11b^+^ population for ErbB4 as well as Ly6C, which marks inflammatory monocytes/macrophages recruited to tissue during inflammation. By the inflammatory phase, a novel population of Ly6C^+^/ErbB4^+^ macrophages emerged in the colons (Figure 5c). The majority of ErbB4^+^ macrophages were Ly6C^+^, as the Ly6C^−^/ErbB4^+^ population was not significantly altered. Furthermore, we detected only low levels of ErbB4 expression on Ly6G^+^ granulocyte/neutrophils and did not see induction on this population during DSS colitis (Figure 5d). Together these results demonstrate that ErbB4 is expressed selectively on inflammatory Ly6C^+^ macrophages recruited to the colon during inflammation.

### NRG4 is repressed by DSS colitis and re-administration reduces macrophage load in the inflamed colon

**Figure 6:**
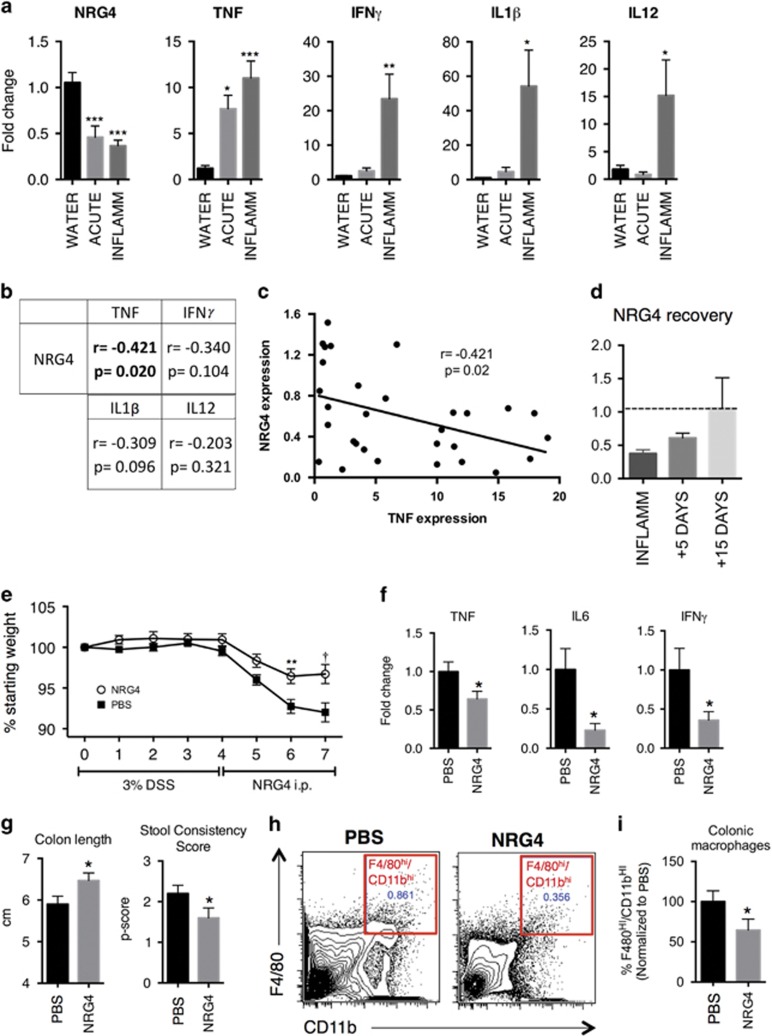
Administration of the ErbB4 ligand NRG4 ameliorates inflammation and reduces colonic macrophage numbers. (**a**) NRG4 and pro-inflammatory cytokine levels in colonic homogenates from mice subjected to acute DSS colitis were determined by qPCR. (**b**) Correlation, *r*, and *P*-value for expression of NRG4 and each cytokine. *n*=10 mice per condition in three independent experiments. (**c**) Correlation plot between relative NRG4 and TNF levels in all mice. (**d**) NRG4 levels were determined in colonic homogenates from mice during recovery (5–15 days post-inflammatory phase) from DSS colitis. Dashed line represents baseline (water controls) expression. (**e**) Weights were recorded daily for mice given 3% DSS in drinking water for 4 days, then removed from DSS and given daily i.p. injections of NRG4 (100 *μ*g/kg). (**f**) At day 7, colonic homogenates were analyzed for macrophage-associated pro-inflammatory cytokine levels by qPCR. (**g**) Colitis parameters were measured at day 7 and (**h**) colonic single cell suspensions were analyzed by flow cytometry for F4/80^HI^/CD11b^HI^ macrophage levels, and quantified (**i**). *n*=10 mice per group. Error bars represent S.E.M. *P<0.05; **P<0.01; ***P<0.001; ^†^*P*<1 × 10^−4^

We have previously shown that in human IBD and chronic mouse colitis, expression of the ErbB4-specific ligand NRG4 is lost, potentially leading to a dysregulated ErbB4 signaling axis.^19^ NRG4 is most prominently expressed in the mesenchyme of the colon,^19^ though Feng and Teitelbaum have also detected expression in epithelium^27^ and we have detected regulated expression in enteroids and immune cells (unpublished results). Thus, NRG4 is likely sourced from multiple cell types in the colon. To determine whether loss of NRG4 in colitis is driven by acute processes early in the injury/inflammation cycle, we analyzed colonic tissue from mice after 4 days of 3% (w/v) DSS exposure (injury phase) and 3 days post DSS (inflammatory phase). NRG4 expression was reduced at the injury phase with further downregulation observed during the inflammatory phase, indicating that NRG4 repression occurs early in colitis and is maintained throughout recruitment of inflammatory macrophages (Figure 6a). As expected, increases in tissue and macrophage-derived pro-inflammatory cytokines TNF, IFN*γ*, IL-1*β*, and IL-12 were observed following DSS treatment (Figure 6a). Previous reports have suggested that pro-inflammatory cytokines may inhibit NRG4 expression in adipocytes^28^ or the intestine.^27, 29^ Consistent with these observations, there was a significant negative correlation (*r*=−0.421; *P*=0.02) between TNF and NRG4 (Figures 6b and c). These observations extend our previous work showing that NRG4 is lost in IBD by showing this inhibition occurs acutely during the initiation of colonic inflammation. Furthermore, colonic NRG4 expression was restored by 15 days post-inflammatory phase (Figure 6d), when colitis is resolving.^30^ These findings suggest NRG4 expression may be suppressed either directly by TNF, or by the same pathogenic processes that induce TNF.

To test if replacing NRG4 during colitis therapeutically alters the macrophage population, mice were given DSS to establish colitis, and then were treated with daily intraperitoneal injections of NRG4 (100 *μ*g/kg) between days 4 and 7, the period of maximal macrophage influx (Figure 5b). NRG4 treatment reversed DSS-induced weight loss (Figure 6e), reduced levels of the macrophage-expressed pro-inflammatory cytokines TNF, IL6, and IFN*γ* (Figure 6f), and ameliorated colon shortening and diarrhea (Figure 6g). Flow cytometric analysis of colonic single cell suspensions for F4/80^HI^/CD11b^HI^ cells showed that, consistent with our *in vitro* observations, NRG4 treatment resulted in a 36% decrease in macrophage numbers in colonic tissue (Figures 6h and i). Thus, when given therapeutically in established acute colitis, NRG4 reduces macrophage numbers in the colon and ameliorates disease.

## Discussion

Dysregulated inflammation is an underlying feature of many chronic diseases, including IBD.^31^ In the intestinal tract, where transient damage and interaction with foreign microbes are frequent, tissue inflammation must initiate rapidly and aggressively to effectively clear a challenge, but must also resolve efficiently to prevent host damage and chronicity. Therefore, anti-inflammatory feedback mechanisms must readily terminate pro-inflammatory responses to maintain homeostasis.^32, 33, 34, 35^ Here, we report the novel finding that ErbB4 signaling provides an example of such a mechanism. This is one of the few known tissue-derived signals promoting resolution of colonic inflammation through pro-inflammatory macrophage death.

Macrophages are crucial mediators of inflammation in the gut. Pro-inflammatory Ly6C^+^ macrophages orchestrate recruitment and activation of adaptive immune responses and aggressively secrete inflammatory factors (TNF, IFN*γ*, IL-1*β*, IL-12) that can result in epithelial damage and loss of barrier function.^2, 36^ Therefore, tight control of these cells is necessary to prevent aberrant or chronic inflammation. In animal models, Ly6C^+^ macrophages recruited from the bloodstream potentiate inflammation, suggesting that overactive responses may contribute to disease.^3, 37^ In contrast, tissue resident macrophage populations (CX_3_CR_1_^+^) integrate and secrete anti-inflammatory signals to prevent colitis and promote tissue repair and resolution.^33, 38^ Identifying subset-specific regulatory mechanisms in macrophage populations is a crucial step toward harnessing these populations therapeutically. Our results show that ErbB4 is a key signaling pathway that limits cell survival specifically in Ly6C^+^ pro-inflammatory, but not naive or anti-inflammatory, macrophages (Figure 2).

Our data show that pro-inflammatory activation of macrophages induces robust ErbB4 receptor expression, and acute colitis is associated with recruitment of ErbB4-expressing macrophages (Figures 1 and 5). In recent years, an increasing number of reports have shown that growth factor receptors, such as the EGFR/ErbB family, FGFRs, and IGF-R, traditionally implicated in regulating epithelial cell function, are also expressed in hematopoietic cell lineages.^39, 40, 41^ To date, however, little is known about what regulates their expression or what role ErbB receptors have in immune cells. Growth factor signals may perform very different functions in immune cells *versus* their established pro-growth and survival functions in epithelium. For example, previous studies have found that phosphorylation of EGFR on macrophages in colitis and gastritis leads to enhanced cytokine release, demonstrating that EGFR signaling may contribute to inflammatory effector functions of these cells.^16, 42^ On the other hand, Tynyakov-Samra and colleagues^14^ found that ErbB4 levels were reduced in PBMCs in patients with the autoimmune disease multiple sclerosis. Furthermore, Ma *et al.*^43^ have observed that gene delivery of NRG4 reduced macrophage markers in liver and adipose tissue, suggesting a possible anti-inflammatory role for ErbB4 signaling in these cells. However, macrophage sub-type expression, a functional role for ErbB4 in macrophage biology, and the expression of ErbB4 in colonic macrophages had not been identified to date.

The finding that ErbB4 signaling in macrophages leads to cell death might seem unexpected, as previous studies with non-transformed cells have largely defined a pro-survival role for this receptor.^19, 44, 45^ However, an apoptotic response to ErbB4 in breast cancer cells has been previously observed, and our findings in macrophages may involve a similar mechanism. In both macrophages (Figure 3) and breast cancer cells,^25^ proteolytic activity appears to be necessary for neuregulin-stimulated apoptosis, likely representing receptor cleavage and generation of the 4ICD intracellular signaling fragment. In NRG4-treated macrophages, both immunostaining and western blot of cellular fractions suggest 4ICD localization to the mitochondria (Figure 3), where mitochondrial-mediated apoptosis may be the mode of action, again consistent with the data from the Jones group. An unanswered question, though, is why this mechanism appears active in some ErbB4-expressing cells (e.g., macrophages, breast cancer) but not others (e.g., colonic epithelial cells). Relative levels of the receptor may have a role, or differential expression of intracellular chaperones regulating localization of 4ICD (cytoplasm *versus* nucleus *versus* mitochondria) may be involved. Furthermore, ErbB4 has several splice variants that can contribute to differences in signaling outcomes.^46^ For example, the cytoplasmic domain variant CYT1 has SH2-binding sites to elicit PI3K signaling, whereas CYT2 variants do not.^47^ Using fibroblasts to compare isoform specific responses, the Elenius group has shown that ErbB4 can both promote either cell survival or cell death under alternative contexts.^48^ Further studies will be necessary to evaluate the relative roles of splicing, environmental context, and other interacting pathways in the signaling outcome in macrophages.

In our experiments, the apoptotic response to ErbB4 activation seems more robust in human M1 macrophages than murine cells. This may be, in part, owing to use of recombinant human NRG4 for these studies, though the human peptide sequence used is 82% identical with mouse (with the majority of the substitutions being conserved for hydrophobicity), and it clearly elicits physiological effects in murine systems *in vitro* (Figures 2 and 3) and *in vivo* (Figure 6). Alternatively, the source of macrophages (bone marrow-derived in the mouse *versus* PBMC-derived from human) may influence as-yet unidentified modifiers of the response. It is possible that future studies comparing the response of human *versus* mouse cells in this context could identify additional molecular pathways selectively targeting pro-inflammatory macrophages and thus altering the balance of innate immune activities in the intestine.

Innate inflammation generated by macrophages has largely been thought of as automatically self-limiting. However, it is becoming clear that resolution of mucosal inflammation is an active process. Thus, understanding the mechanisms that resolve inflammation has emerged as a key question in this field. Mechanisms driving clearance of macrophages are not well understood. In chronic inflammation, these cells are continually replenished by recruitment from circulation, which can create a feed-forward loop. One aspect that may contribute to chronic inflammation is a failure of appropriate self-termination of these cells.^49^ In colitis, NRG4 expression is inhibited,^19^ leading to an incomplete or altered ErbB4 signaling circuit. Here, we extended our previous findings by showing this loss of NRG4 occurs early in a model of acute colitis, and NRG4 levels are negatively correlated with TNF expression (Figure 6). Intriguingly, replacement of lost NRG4 with exogenous administration following induction of injury in DSS colitis to complete this circuit significantly attenuated inflammation and reduced colonic macrophage numbers (Figure 6). ErbB4 expression was undetectable on murine BMDCs and neutrophils (Figure 1), and minimally on neutrophils collected from inflamed mouse colons (Figure 5d), or human PBMC-derived neutrophils (Figure 4e). As macrophages are the only myeloid cells on which we observed robust ErbB4 expression, together with our NRG4 rescue experiment results these findings support the idea that ErbB4 signaling in macrophages is a selective anti-inflammatory feedback mechanism in colitis. It is possible that downregulation of NRG4 may be necessary for a maximal innate immune response to a challenge. Defects in re-expression of this ligand, which occurs coincident with recovery in DSS colitis (Figure 6d), may contribute to chronic inflammation. Future studies to identify the key cellular source of NRG4 in the colon and how its expression is regulated will provide insight into a possible mechanism underlying chronic colitis. Furthermore, as macrophages are early responding cells that shape the development of the immune response, long-term *in vivo* studies will be required to understand how ErbB4 loss in macrophages impacts adaptive immunity and potentially alters local factors, such as the microbiota, in ways that may be involved in colitis development and resolution.

In summary, our data indicate that ErbB4 is induced on macrophages during inflammation as a mode of feedback inhibition. ErbB4 activation elicits clearance of inflammatory macrophages and promotes recovery. Administration of exogenous NRG4, or identifying other methods to activate ErbB4 signaling in these cells, is a potential approach to alleviate disease in IBD patients and other patients with chronic, macrophage-dependent inflammation. Furthermore, the opposing roles for ErbB4 signaling in different cell types – supporting epithelial survival^17, 19, 50^ while triggering macrophage apoptosis – suggest a coordinated pro-recovery role for ErbB4 signaling in the face of injury and inflammation.

## Materials and methods

### Animal experiments

All animal use was approved and monitored by the Children's Hospital Los Angeles Institutional Animal Care and Use Committee (Animal Welfare Assurance #A3276-01). Mice were housed under standard conditions with *ad libitum* water and chow access in the AAALAC-accredited animal care facility at Children's Hospital Los Angeles. C57Bl/6 mice obtained from Jackson Laboratory aged 8–12 weeks were used for experiments. For acute colitis, mice were given 3% (w/v) DSS in drinking water for 4 days (injury phase), followed by 3 days without drinking water (inflammatory phase). For NRG4 recovery analysis, colons were collected at 5 and 15 days post-inflammatory phase (recovery and repair). Stool scores were recorded on a scale of 0–4 as previously described on a continuum from fully formed pellets at 0 to liquid stool at 4.^51^

### Bone marrow macrophage and dendritic cell culture

Isolated bone marrow from mice was incubated with filtered CMG14–12 conditioned media (1 : 20) containing M-CSF to generate BMDM as previously described,^52, 53, 54^ or 20 ng/ml GM-CSF (Thermo Scientific, Waltham, MA, USA, PMC2015) to generate BMDC as previously described.^55^ For BMDMs, adherent cells were washed at 3 days and re-fed with M-CSF containing media until experimentation at day 7–8. For M1 polarization, cells were pre-treated with 100 U/ml IFN*γ* for 16 h, then stimulated with 100 ng/ml LPS from *Escherichia coli* 0111:B4, purified by gel-filtration chromatography (Sigma, St. Louis, MO, USA, cat# L3012). For M2 polarization, cells were stimulated with 10 ng/ml IL-4 (Gibco, Waltham, MA, USA, PMC0046). For BMDCs, cells were re-fed with GM-CSF at day 3 of culture. After 7 days, loosely adherent cells (enriched dendritic cell population) were transferred to new plates for experimentation. Neutrophils were isolated from bone marrow of C57BL/6 mice by Percoll density gradient separation as previously described.^56^ In brief, 100% Percoll (GE Life Sciences, Pittsburgh, PA, USA, 17-0891-01) (9 parts Percoll:1 part PBS) was diluted to 78%, 69%, and 52% solutions using PBS and layered in 5 ml tubes with bone marrow on the uppermost layer. After a 30 min centrifugation at 1500 g, the layer of cells at the 78%/69% interface was isolated, treated with or without 10 ng/ml GM-CSF (Life Techonologies, Carlsbad, CA, USA, PMC2015), and used in subsequent studies.

### Immunofluorescence staining

BMDMs grown on coverslips were fixed with ice-cold acetone for 30 min, blocked with 10% goat serum for 1 h at room temperature, and incubated with 1 : 200 primary antibody against ErbB4 (Santa Cruz, Dallas, TX, USA, sc-283) overnight. Antigenic peptide competition controls were performed to confirm specificity. Cells were washed and incubated with 1 : 1000 secondary rabbit anti-mouse Alexafluor-555 (Life Technologies) for 1 h at room temperature following by mounting with Vectashield mounting media including DAPI (Vector Labs, Burlingame, CA, USA, H-1500).

### Real-time PCR

RNA from cells and tissue was collected using on-column RNA isolation and purification (OMEGA Biotek, Norcross, GA, USA), and cDNA generated with a high-capacity cDNA reverse transcriptase kit (Applied Biosystems, Foster City, CA, USA, 4368814). Quantitative analysis of expression was performed using TaqMan assays (EGFR (Mm01187858_m1), ErbB2 (Mm00658541_m1), ErbB3 (Mm01159999_m1), ErbB4 (Mm01256793_m1), NRG4 (Mm00446254_m1), IFNγ (Mm01168134_m1), IL-1*β* (Mm00434228_m1), TNF (Mm00443258_m1), IL6 (Mm00446190_m1), and IL-12 (Mm00434169_m1), HPRT (Mm03024075_m1)) on an Applied Biosystems StepOne thermocycler. Fold change was calculated using the 2^−ΔΔCt^ method.^57^ Results are expressed as average fold change in gene expression relative to control or non-treatment group using HPRT as the reference gene.

### Western blotting

Protein lysates from cells and tissue were collected and lysed in RIPA buffer^17^ with Halt Protease inhibitor cocktail (Thermo Scientific, #1861278), and phosphatase inhibitor cocktails 2 and 3 (Sigma, P5726 and P0044). Mitochondria were isolated according to the manufacturer's protocol using the Mitochondrial Isolation Kit for Cultured Cells (Thermo Scientific, PI89874). Protein concentration was determined by DC protein assay (Bio-Rad, Hercules, CA, USA, #500). Thirty microgram protein/condition were separated by SDS-PAGE (Thermo Scientific, NW0412A) and transferred to nitrocellulose membrane. Membranes were blocked with 5% milk and probed with 1:1000 EGFR (Cell Signaling, Danvers, MA, USA, #4267), 1:1000 ErbB2 (Cell Signaling #2165), 1:1000 ErbB3 (Cell Signaling #12708), 1 : 1000 rabbit anti-ErbB4 (Santa Cruz, sc-283) overnight at 4 °C or 1:10 000 mouse anti-Actin (Sigma, A1978) for 1 h at room temperature, followed by 1:10 000 IRDye-conjugated donkey anti-rabbit (LI-COR, Lincoln, NE, USA, #926–68023) and donkey anti-mouse (LI-COR, #926–32212) for 1 h at room temperature and quantification on an Odyssey imager (LI-COR).

### Murine cell viability and apoptosis assays

BMDMs were plated in 96-well plates at 40 000 cells/well. Cells were washed and plated in DMEM with 10% heat-inactivated FBS, 100 U/ml penicillin and streptomycin, and given 100 U/ml IFN*γ* overnight. In some experiments cells were then pre-treated for 30 min with 2 *μ*g/ml ErbB4 neutralizing antibody (Millipore, Billerica, MA, USA, 05–478)^58^ before incubation with 100 ng/ml NRG4 (Reprokine, Valley Cottage, NY, USA) for 1 h then LPS (100 ng/ml) for 48 h. Some cells were pre-treated with the metalloprotease inhibitor GM6001 (Tocris, Minneapolis, MN, USA, #2983) at 10 *μ*M, *γ*-secretase inhibitor DAPT (Tocris, #2634) at 10 *μ*M, ADAM17 inhibitor GW280264X at 3 *μ*M (Aobious, Inc., Gloucester, MA, USA), RIPK1 inhibitor, Necrostatin-1 at 50 *μ*M (Cayman Chemical, Ann Arbor, MI, USA, 11658), or caspase-9 activation inhibitor, NS3694 at 10 *μ*M (Millipore, 178494) prior to NRG4 and LPS treatment as described in Results. Relative cell numbers were determined by Cell Titer Blue (resazurin-based assay) following manufacturer's instructions (Promega, Madison, WI, USA, G8081). For active caspase-3 analysis in BMDMs, cells were fixed with ice-cold acetone for 30 min and incubated overnight at 4 °C with antibody against cleaved caspase-3 pre-conjugated to Alexa Fluor 488 (Cell Signaling, #9669). The following day, cells were washed 5 × with PBS for 5 min each and imaged. We performed analysis of membrane phosphatidylserine flip with annexin V staining. Following treatment, cells were stained with annexin V and propidium iodide using the annexin V apoptosis kit (Life Technologies, V13241) and analyzed on an LSR II flow cytometer (BD Biosciences, San Jose, CA, USA). To assess mitochondrial transmembrane potential, MitoCapture mitochondrial apoptosis detection kit (ENZO Life Sciences, Farmingdale, NY, USA, ALX-850-232) was used according to the manufacturer's protocol. Briefly, cells were incubated for 20 min with MitoCapture dye, then analyzed by flow cytometry. Healthy cells are labeled in the red channel and cells unable to take up dye in the mitochondria (due to altered mitochondrial transmembrane potential) are detectable in the green channel.

### Primary human myeloid cell culture, MDM polarization, and cell survival

Informed consent was obtained per protocol approved and monitored by the institutional review board at Yale University. We recruited healthy individuals with no personal or family history of autoimmune/inflammatory disease, including psoriasis, SLE, rheumatoid arthritis, multiple sclerosis, type I diabetes mellitus, Crohn's disease, and ulcerative colitis, or a history of HIV. Human PBMCs were isolated using Ficoll-Paque (GE Dharmacon, Lafayette, CO, USA). In some experiments, cells were stained using CD11b (BD Biosciences, 555388) and CD15 (BD Biosciences, 555401). Monocytes were purified from PBMCs by positive CD14 selection (Miltenyi Biotec, Auburn, CA, USA) or adhesion, tested for purity, and cultured with M-CSF (10 ng/ml) (Shenandoah Technology, Woodstock, VA, USA) for 7 days for MDM differentiation in the presence of 10% fetal bovine serum (Sigma). MDMs were stimulated with 100 ng/ml LPS (Sigma) and 20 ng/ml IFN-*γ* (R&D Systems, Minneapolis, MN, USA) (M1 polarization) or 20 ng/ml IL-4 (R&D Systems) (M2 polarization), with or without NRG4. Where indicated, cultures were transfected with 100 nM scrambled or ON-TARGETplus SMARTpool siRNAs (a pool of four distinct, commercially designed siRNA) against ErbB4 (GE Dharmacon) using Amaxa nucleofector technology (Amaxa, San Diego, CA, USA). Apoptosis was detected by flow cytometry with annexin V (eBiosciences, Waltham, MA, USA). Intracellular proteins were detected in permeabilized cells by flow cytometry with anti-ErbB4 (Abcam, San Francisco, CA, USA, ab32375).

### Flow cytometry

To generate a single cell suspension, colonic mucosa was isolated and digested for 30 min at 37 C in 100 ml DMEM with 2% heat-inactivated FBS, 0.2 mg/ml dispase II (Sigma, D4693), 2 mg/ml collagenase D (Roche, Indianapolis, IN, USA, #11088882001), and 0.2 mg/ml DNase I (Sigma, DN25) as previously described.^59^ For population analysis experiments, cells were fixed with 4% formaldehyde followed by permeabilization with 0.01% saponin. Cells were incubated with FcBlock (BD Biosciences, 553142, 1:100) for 15 min in FACS buffer (PBS+1% heat-inactivated FBS), followed by incubation for 30 min with the following fluorophore pre-conjugated antibodies: Ly6G FITC (Life Technologies, A25990, 1:100), F4/80-Alexafluor 488 (Life Technologies, MF48020, 1:100), CD11b-APC (Life Technologies, RM2805, 1:100), and Ly6C-BV421 (BD Biosciences, 562727, 1:100). Cells were also incubated with primary antibody against monoclonal ErbB4 (Abcam, ab32375, 1:40) for 30 min followed by anti-rabbit PE (Abcam, A10542, 1:100) for 30 min. Cells were analyzed on an LSR II flow cytometer (BD Biosciences).

### Statistical methods

Statistical analyses and plots were generated using Prism (GraphPad Software). Mean±S.E.M. is depicted in bar graphs. Student's *t-*test or ANOVA with Tukey *post hoc* test to correct for multiple comparisons were used to determine statistical differences, as appropriate. Statistical significance was assigned to *P*<0.05.
